# Synthesis, Photo-Physical Properties, and Electroluminescence Characteristics of Iridium Phosphorescent Materials Based on Different β-Diketonate Ancillary Ligands

**DOI:** 10.3390/molecules30040861

**Published:** 2025-02-13

**Authors:** Qiaowen Chang, Ke Zhang, Caixian Yan, Liming Xie, Yuanqiuqiang Yi, Wenming Su, Weiping Liu

**Affiliations:** 1 State Key Laboratory of Precious Metal Functional Materials, Yunnan Precious Metals Laboratory, Kunming Institute of Precious Metals, Kunming 650106, China; zhangke120612@163.com (K.Z.); ycx19860706@163.com (C.Y.); liuweiping0917@126.com (W.L.); 2 Printable Electronics Research Center, Nano-Devices and Materials Division, Suzhou Institute of Nano-Tech and Nano-Bionics, Chinese Academy of Sciences, Suzhou 215123, China; lmxie2014@sinano.ac.cn (L.X.); wmsu2008@sinano.ac.cn (W.S.); 3 Joint International Research Laboratory of Information Display and Visualization, School of Electronic Science and Engineering, Southeast University, Nanjing 210096, China; 4 Gusu Laboratory of Materials, Suzhou 215123, China

**Keywords:** iridium (III) complex, ancillary ligand, deep-red emitter, OLED, phosphorescent materials

## Abstract

Organic light-emitting diodes (OLEDs) based on phosphorescent materials are among the most promising technologies for displays and lightings. For red-emitting heteroleptic iridium complexes (HICs), vast and major research has been focused on the design and synthesis of cyclometalated ligands, while relatively little attention has been given to ancillary ligands which also play important roles in manipulating the optoelectronic and electroluminescent properties of HICs. Seven deep red-emitting HICs were designed and synthesized by systematically modifying the alkyl groups in β-diketone-type ancillary ligands. These HICs exhibited similar physical and optoelectronic properties, with OLED devices based on these materials achieving consistent emission peaks at 624 nm and CIE coordinates of (0.68, 0.32). Among the synthesized HICs, Ir(dmippiq)₂(dmeacac), featuring 3,7-dimethyl-4,6-nonanedione as the ancillary ligand, demonstrated the best OLED performance, achieving a champion external quantum efficiency (EQE) of 18.26%. This result highlights that engineering the alkyl groups in β-diketone ancillary ligands can significantly enhance device performance.

## 1. Introduction

With unique features of fast response, flexibility, high efficiency, high brightness, and so forth, organic light-emitting diodes (OLEDs) have been one of the most competitive technologies in display markets [1,2] and future energy-saving solid-state lighting sources [3,4,5,6].

OLEDs based on phosphorescent materials can harvest both singlet and triplet excitons to achieve a near theoretically 100% internal quantum efficiency [7,8,9]. Among various phosphorescent materials containing heavy metals [10], the Ir(III) complexes have been deemed as the most successful species for OLEDs since iridium green and red emitters have been successfully commercialized [11,12,13,14].

Although red iridium phosphorescent materials have been commercially applied, the pursuit of higher efficiency and superior color purity continues to be a focus of both academia and industry, driven by the growing demand for high-definition displays. Although extensive efforts have been devoted to obtaining highly phosphorescent Ir(III) complexes with deep-red emission, achieving high-color purity and high efficiency meanwhile remains a great challenge [15,16,17,18,19]. Heteroleptic iridium phosphorescent complexes with moderately strong emission at room temperature are widely researched in highly phosphorescent iridium complexes with red emission. For heteroleptic iridium phosphorescent complexes, the emission properties can be influenced by chemical structure and electronic properties of the cyclometalated ligands and the ancillary. Generally, it is considered that the emission properties of the phosphorescent iridium complexes are affected prominently by the cyclometalated ligands. However, it is noteworthy that the emission properties can also be influenced by the ancillary ligands. Therefore, unveiling the role of the ancillary ligands is also significant for designing excellent phosphorescent materials. For heteroleptic iridium complexes (HICs), the cyclometalated ligands (C^^^N) together with the ancillary ligands β-diketone (O^^^O) have great influences on the electroluminescence properties [20,21,22]. The variations of ancillary ligands not only influence the photoluminescence quantum yield and electroluminescence properties, but also affect the thermal stability, film formation ability, and charge carrier transport ability [23]. As a commonly used O^^^O-type bidentate ancillary ligand, various β-diketones can coordinate with Ir(III) to form stable octahedral complexes and regulate their triplet energy levels to influence the phosphorescent emission spectrum [24,25,26,27,28]. For red-emitting HICs, it is normally required to select β-diketone as ancillary ligands to achieve high triplet energy levels, such as 3,7-diethylnonane-4,6-diones, which can improve the color purity and significantly lower the sublimation temperature [20]. Even though the acetylacetone and 2,2,6,6-tetramethyl-3,5-heptanedione are the most widely-used ancillary ligands in red-emitting HICs [28,29,30,31,32,33,34], other β-diketone-based HICs are barely investigated and there is no clear structure–property relationship between them [35]. As such, designing and synthesizing different β-diketone-based HICs and studying the structure–property relationship can provide an insight into the influence of ancillary ligands on the optoelectronic and luminescent properties of HICs.

In this study, we designed and synthesized seven red-emitting HICs by modifying the chemical structure of β-diketone ancillary ligands. Our findings reveal that changes in the chemical structure of the β-diketone ancillary ligands have negligible effects on the fundamental physical and optoelectronic properties. Consequently, the series of HICs exhibited comparable UV–vis absorption and fluorescence spectra. However, slight modifications to the chemical structure of β-diketone ancillary ligands can significantly enhance the electroluminescence performance of iridium-based phosphorescent materials. Ir(dmippiq)_2_(dmeacac) with 3,7-dimethyl-4,6-nonanedione as an ancillary ligand exhibited a champion OLED device performance with an EQE of 18.26%, which is much higher than that of the other six HIC-based PhOLEDs.

## 2. Results and Discussion

### 2.1. Synthesis and Thermal Properties

**Scheme 1:**
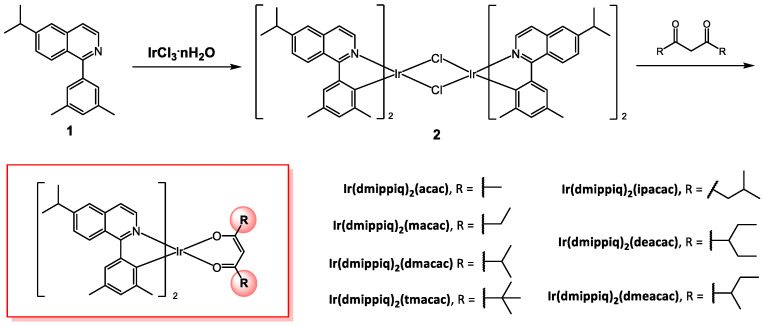
Chemical structures of HICs and corresponding synthetic routes.

To evaluate the effect of β-diketone ancillary ligands on the performance of HICs, seven deep red-emitting HICs were designed and synthesized with different lengths and branch points. Subsequently, ligand **1** reacted with IrCl_3_·nH_2_O to obtain compound **2** [36]. Then, various β-diketones with different alkyl substitutes reacted with compound **2** to deliver the seven red HICs, as illustrated in Scheme 1.

**Figure 1:**
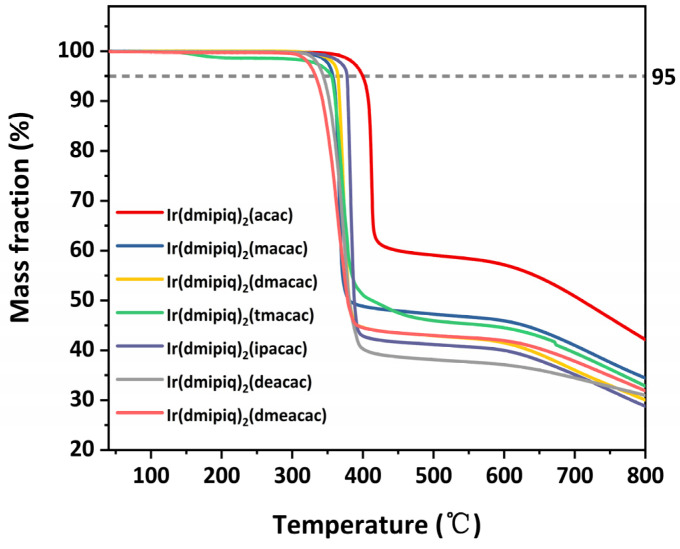
TGA curves of the seven red-emitting HICs.

On the working conditions of PhOLEDs, it is inevitable that joule heat would be produced and thus in some degree cause damages to the organic functional thin film, which requires that the materials used should have enough good thermal stability. By employing thermal gravimetric analysis (TGA) measurements as shown in Figure 1, the seven red HICs with different β-diketone all show high thermal decomposition temperature (T_d_, corresponding to 5% weight loss) above 330 °C, and Ir(dmipiq)_2_(acac) shows an especially comparatively high T_d_ of 400 °C. These results indicate that they all have enough thermal stability for fabrications of efficient stable OLEDs.

### 2.2. Crystalline Structures

**Figure 2:**
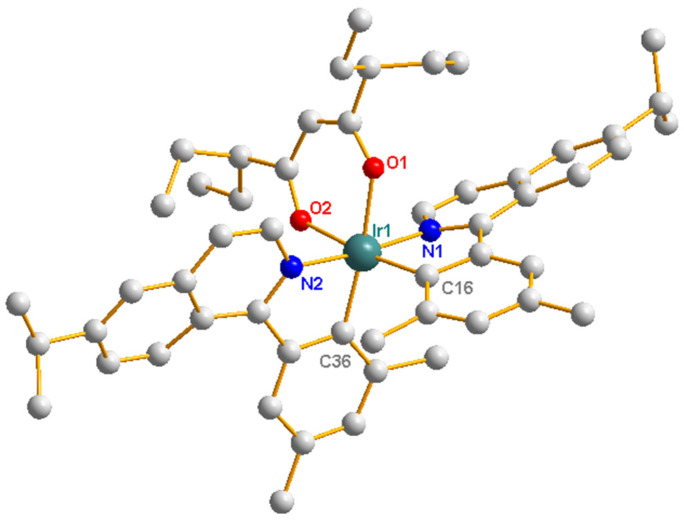
Crystal structure of Ir(dmippiq)_2_(deacac) (note: hydrogen atoms are omitted).

The crystal structure of Ir(dmippiq)_2_(deacac) is shown in Figure 2, and the partial crystal data of the crystal are shown in Table 1 and Table 2. There are two molecules in the crystal structure. The iridium phosphorescent complexes exhibit a slightly distorted octahedral configuration. The C and N atoms involved in the coordination of the main ligand formed a coordination angle of about 80° with the central Ir atom. The two O atoms in the auxiliary ligand formed a five-membered ring chelate with the central Ir atom, with a coordination angle of about 87°. The central metal iridium (III) chelates and coordinates with the C and N atoms of two main ligands to form two stable pentagonal ring and with the two O atoms of acetylacetone to form one stable hexagonal ring. The two coordinating nitrogen atoms in the main ligand are in trans conformation, and the two coordinating carbon atoms are in cis conformation.

### 2.3. Optical and Electrochemical Properties

**Figure 3:**
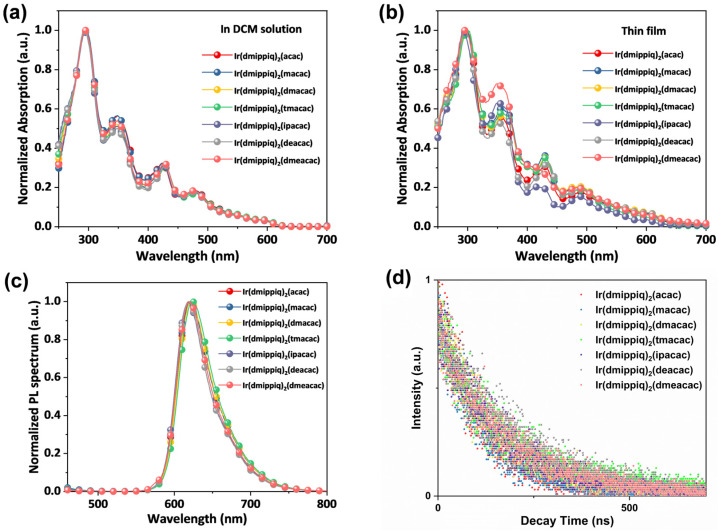
(**a**) UV–vis absorption, (**b**) the thin film UV–vis absorption and (**c**) emission spectra, (**d**) phosphorescent lifetime of the seven red-emitting HICs.

The ultraviolet visible (UV–vis) absorption and emission spectra at room temperature were measured to study the photophysical characteristics of the seven red HICs. As shown in Figure 3a,c and summarized in Table 3, the series compounds exhibit similar UV–vis absorption spectral features in diluted dichloromethane (DCM) solutions. The two strong absorptions in the ultraviolet and visible region of 290–350 nm of the series compounds can be attributed to intra-ligand (C^^^N) ^1^π-π* absorption [37,38,39]. There are two weaker and broader absorptions on the shoulders in the range of 400–500 nm, which can be ascribed to spin-allowed and spin-forbidden metal-to-ligand transfer (^1^MLCT and ^3^MLCT) transitions of the Ir(III) phosphorescent emitters [24]. The weakest absorption around the 590–600 nm region can be attributed to the triplet charge transfer of the ligand (^3^LC).

Since the PhOLEDs are thin-film devices, the thin film states are crucial to the device performance. The thin film UV–vis absorption of the seven HICs are also further measured to evaluate the effect of different alkyl groups in ancillary ligands of β-diketones. As depicted in Figure 3b and compared to Figure 3a, all the seven HICs give enhanced absorptions on the shoulders, especially the absorption peaks around 350 nm. Ir(dmipiq)_2_(dmeacac) shows a comparatively higher absorption peak at 350 nm, indicating its more ordered molecular interactions in the thin film state, which is in favor of its comparatively higher device performance as discussed later.

The emission spectrums of the seven HICs in DCM solution are shown in Figure 3c. As observed, the maximum emission peaks of the series compounds are close in the region of 618–624 nm and the full widths at half maximum (FWHM) are of the same value of 51 nm, demonstrating that the ancillary ligands of β-diketone have little influences on the absorption and emission spectrum. These results can be mainly attributed to that the triplet energies of the seven β-diketone used in the deep red-emtting phosphorescent materials are much higher than those of cyclometalated ligand. The solution are degassed by bubbling nitrogen 3 min for measuring the photoluminescence quantum yields and emission decay curves. The photoluminescence quantum yields (PLQYs) of these emitters measured in de-oxygen DCM solutions are between 66% and 75%. It is worth noting that Ir(dmippiq)_2_(acac), Ir(dmippiq)_2_(macac), Ir(dmippiq)_2_(tmacac), and Ir(dmippiq)_2_(dmeacac) exhibit PLQYs over 70%, indicating their practical potentials as efficient emitters in PhOLEDs. The seven HICs display comparable emission decay curves, characterized by excited-state lifetimes within the microsecond range (as depicted in Figure 3d). This observation suggests that the emission originates predominantly from the triplet excited state. Notably, shorter phosphorescence lifetimes decrease the probability of triplet–triplet annihilation (TTA) and triplet–polaron annihilation (TPA) processes. Across all seven HICs, the radiative decay rate constant (k_r_) surpasses the non-radiative decay rate constant (k_nr_). This superiority can be attributed to the substantial contribution of the metal-to-ligand charge transfer (MLCT) state to the triplet excited state (T1), which enhances spin-orbit coupling and consequently leads to a larger k_r_. The combination of a relatively high k_r_ and a short excited-state lifetime indicates that triplet excitons undergo efficient decay through radiative transitions. This rapid decay mechanism is pivotal for the advancement of highly efficient OLED devices.

### 2.4. Electrochemical Properties

**Figure 4:**
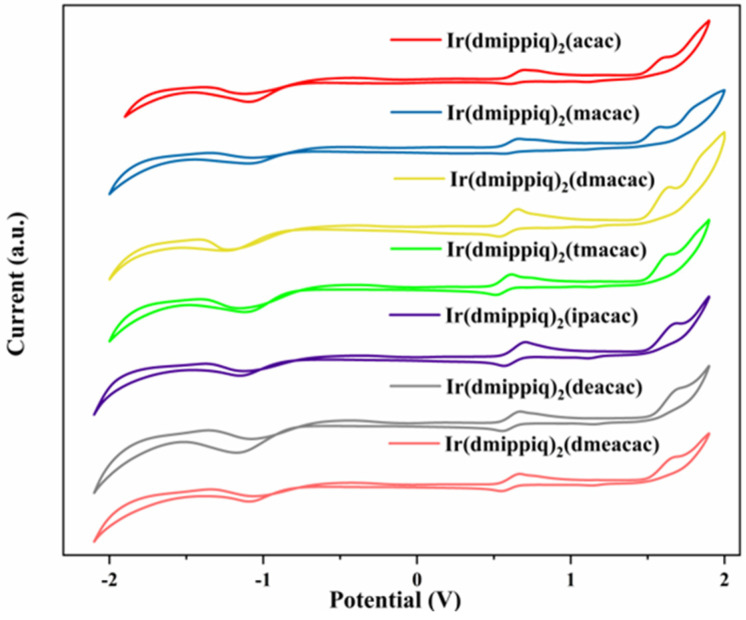
Cyclic voltammograms for the seven HICs.

Using ferrocene as the benchmark and silver/silver chloride as the reference electrode, a 0.1 mol/L dichloromethane solution was prepared with tetrabutylammonium hexafluorphosphate serving as the electrolyte. Subsequently, a precise quantity of seven HICs was introduced into the solution to attain a sample concentration of 0.001 mol/L. The electrochemical characteristics of these seven HICs were then rigorously examined (Figure 4). The cyclic voltammetry curves of the seven HICs exhibited remarkably similar variations, featuring a reversible oxidation peak centered around 0.5 V. This particular oxidation peak is attributed to the Ir^3+^/Ir^4+^ transition at the core of the HICs. Leveraging the oxidation onset potential observed in the curves and utilizing the empirical formula E_HOMO_ = −e(E_ox_ + 4.4) eV for electrochemical calculations, the HOMO (Highest Occupied Molecular Orbital) energy levels of the seven HICs were determined. Furthermore, the energy gaps (E_g_) of the HICs were calculated from their absorption spectra using the formula E_g_ = 1240/λ. By employing the relationship E_LUMO_ = E_g_ + E_HOMO_, the LUMO (Lowest Unoccupied Molecular Orbital) energy levels of the seven HICs were derived. These calculated values are comprehensively summarized in Table 4. Notably, the seven HICs exhibited very similar HOMO/LUMO levels and energy bandgaps, which are in excellent alignment with their absorption spectra.

### 2.5. Theorectical Calculations

**Figure 5:**
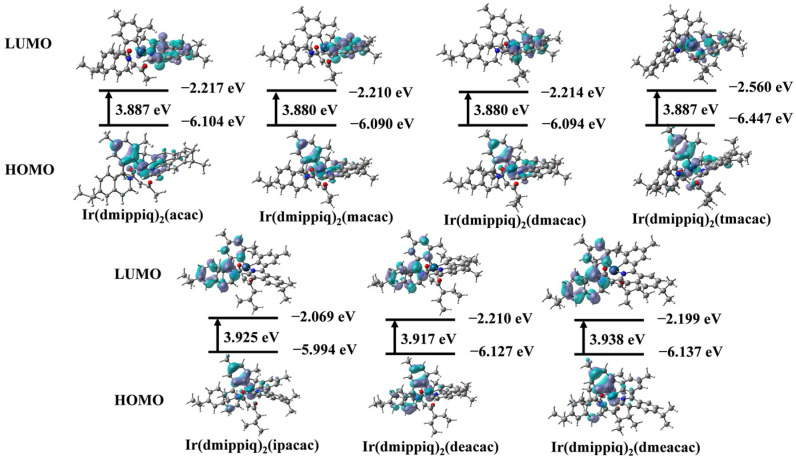
Distribution of calculated frontier molecular orbitals and HOMO-LUMO(H-L) energy gaps for HICs.

Computational investigations have been proven to be very helpful in understanding the HOMO and LUMO properties of cyclometalated iridium phosphorescent complexes. To better understand the fundamental properties of the seven HICs, the density functional theory (DFT) calculations were conducted, and the optimized HOMO/LUMO distributions and energy gaps are illustrated in Figure 5. All seven HICs have similar energy gaps from 3.880 eV to 3.938 eV; the small variation in energy gaps indicates that the substitution or modifications of the ligands in these HICs do not drastically alter their electronic structure, which, consistent with the experimental results of seven HICs, have similar photoluminescence spectra and emission wavelengths.

Interestingly, the HOMO and LUMO energy level distribution are significantly different. The Ir(dmippiq)_2_(acac), Ir(dmippiq)_2_(macac), Ir(dmippiq)_2_(dmacac), and Ir(dmippiq)_2_(tmacac) have similar HOMO and LUMO energy level distribution, the HOMO energy level is primarily localized on the metal and the benzene ring of the cyclometalated ligand, while the LUMO energy level is mainly localized on the isoquinoline ring. However, the other three HICs have different energy level distributions. The HOMO energy level is primarily localized on the metal and the benzene ring of the cyclometalated ligand, while the LUMO energy level is mainly localized on the benzene ring and isoquinoline ring. Especially, the LUMO electron cloud distribution of Ir(dmippiq)_2_(dmeacac) is more dispersed and farther away from the iridium center. This may lead to a decrease in the triplet energy level and improving the material’s electron mobility and antioxidant capacity, which has potential to become a candidate for OLED materials.

### 2.6. EL Device Performance

**Figure 6:**
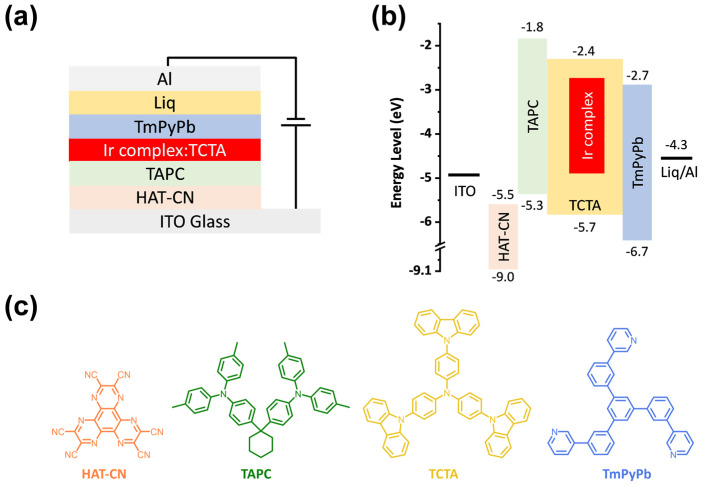
(**a**) Device structure of PhOLEDs, (**b**) energy levels, and (**c**) molecular structures of each materials used.

To evaluate the alkyl group effect of β-diketone in HICs for PhOLEDs, the multilayered PhOLEDs with a device configuration of ITO/HAT-CN (5 nm)/TAPC (30 nm)/Ir(III) complex doped in TCTA(30 nm)/TmPyPb (30 nm)/Liq (2 nm)/Al (100 nm), where HAT-CN, TAPC, TCTA, TmPyPb and Liq are referred to as 2,3,6,7,10,11-hexacyano-1,4,5,8,9,12-hexaazatriphenylene, 1,1-bis(4-bis(4-methylphenyl)aminophenyl)cyclohexan-e, tris(4-carbazoyl-9-ylphenyl)amine, 3,3′-(5′-(3-(3-pyridinyl)phenyl)(1,1′:3′,1′′-terphenyl)-3,3′′-diyl)bispyridine and 8-hydroxyquinolinolato-lithium, respectively. The energy level diagram and the molecular structures of the materials used in the PhOLEDs are as shown in Figure 6.

**Figure 7:**
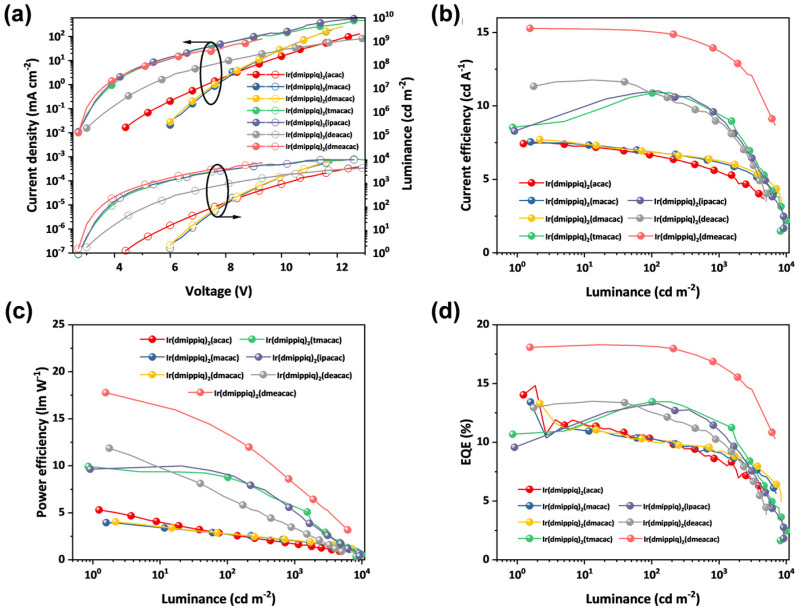
(**a**) Current density–voltage–luminance (*J*-*V*-*L*) characteristics, (**b**) current-efficiency (CE) versus luminance curves, (**c**) power efficiency (PE) versus luminance curves, and (**d**) EQE values versus luminance curves of the seven emitters based their optimized PhOLEDs.

From the point of view of the energy level matching, TCTA was chosen as the host material. PhOLEDs based on the seven HICs with different β-diketones are systematically optimized by variations of each compound with different doping concentrations (Figure 7 and Table 5). As summarized in Table 5, the seven HIC-based deep red-emitting PhOLEDs realized the single EL peak in a range of 624–626 nm, without emission from the hosts or other species, indicating that the energy can be totally transferred from the fluorescent host to the Ir(III) dopants. The series PhOLEDs gave Commission Internationale del Eclairage (CIE) coordinates of the same value of (0.68, 0.32) and all delivered a saturated red emission color, further demonstrating that the β-diketone almost has no impact on the EL wavelengths.

The optimal doping concentrations of all the HICs were in a range of 10–12%, except for Ir(dmippiq)_2_(dmeacac) with 16% considering its bulky substitute and large molecular weight. The comparatively high doping concentrations of these complexes illustrate that these molecules are not easy to form molecular packing, which would cause serious aggregations and concentration quenching effects. The PhOLED based on Ir(dmippiq)_2_(dmeacac) achieved the best device performance, with a maximum luminance (L_max_) of 13,960 cd/m^2^, a maximum current efficiency (CE_max_) of 15.63 cd/A, a maximum power efficiency (PE_max_) of 12.60 lm/W. In addition, PhOLED based on Ir(dmippiq)_2_(dmeacac) exhibited a maximum external quantum efficiency (EQE_max_) of 18.26%, which is much higher than that of devices based on the other six HICs (only with around 13% EQE_max_). It is worth noting that the turn-on and working voltage at 100 and 1000 cd/m^2^ of the device based on Ir(dmippiq)_2_(dmeacac) is as low as 2.6, 3.6, and 5.4 V, which is beneficial for device working stability.

More importantly, Ir(dmippiq)_2_(dmeacac) harvests better device performance than the commercially available Ir(dmippiq)_2_(deacac), revealing its great potentials as a deep-red phosphorescent emitter in OLED displays.

## 3. Materials and Methods

### 3.1. General Information

All the commercially available materials and solvents for device fabrications were purchased from Sigma-Aldrich (Shanghai) Trading Co., Ltd., J&K Scientific, Suna Tech Inc., Shanghai Hanfeng Co. (Shanghai, China) and used as received without purifications unless otherwise stated. All reactions were performed under an argon atmosphere unless otherwise stated. ^1^H and ^13^C NMR spectra were recorded on a Bruker AV-500 spectrometer (Brucker, Berlin, Germany) at room temperature. Elemental analysis was performed with the Elemental Vario EL III instrument (ELEMENTAR, Hanau, Germany). Cyclic voltammetry (CV) was carried out using a CH Instrument 660C electrochemical analyzer (Shanghai CH, Shanghai, China) with a Ag/Ag^+^ electrode as a reference electrode and tetra (n-butyl)ammonium hexafluorophosphate (TBAPF_6_) as a supporting electrolyte. UV–vis absorption spectra were recorded on a Perkin-Elmer Lambda 750 (PerkinElmer, Waltham, MA, USA). The absolute PLQY was determined using a Quantaurus-QY Absolute photoluminescence quantum yield spectrometer (C11347-11, Hamamatsu Photonics, Hamamatsu, Japan). The decay curves were characterized by Edinburgh FLS 1000 by bubbling the nitrogen for 3 min.

### 3.2. Device Fabrication and Measurement

Before use, the pre-patterned ITO glass was washed with glass cleaner, deionized water, and absolute ethanol, and treated with oxygen plasma for 3 min. The PhOLEDs based on the seven HICs are fabricated by vacuum deposition of the organic layer and cathode onto a pre-patterned indium–tin–oxide (ITO) glass substrate (2 × 2 mm pixels) in a vacuum chamber in the vacuum of 1.5 × 10^−6^ Torr. For the entire process of organic functional layers, LiF and aluminum cathode were vacuum deposited without exposure to the atmosphere. The thicknesses of each film was measured using an Alpha Step profilometer (Dektak150, Veeco Instruments Inc., Plainview, NY, USA). The devices were encapsulated using ultraviolet-curing adhesives and cover glass. All electrical testing and optical measurements were performed under ambient conditions. The electroluminescent spectrum was measured with a Spectra Scan PR655 (JADAK, North Syracuse, NY, USA). The current–voltage (*J*-*V*) and luminance–voltage (*L*-*V*) relations were characterized with a computer-controlled Keithley 2400 Sourcemeter (Tektronix, Beaverton, OR, USA).

### 3.3. Compound Synthesis

To a three-neck round bottle (1 L), IrCl_3_⸱nH_2_O (24 g, 67.42 mmol) and 3,5-dimethylphenyl-6-isopropyl-isoquinoline 1 (50.00 g, 181.56 mmol) were added. Subsequently, 2-methoxyethanol (450 mL) and H_2_O (150 mL) was added into the mixture. The reaction mixture was stirred to reflux at 100 °C for 24 h. After cooling to room temperature, the reaction mixture was filtered and the solid cake was washed with deionized water, anhydrous ethanol, and hexane, sequentially. Deep-red solid compound 2 (44.50 g) was obtained with a yield of 97.01%.

Ir(dmippiq)_2_(acac): To a three-neck round bottle (500 mL), compound 2 (5 g, 3.67 mmol), anhydrous Na_2_CO_3_ (1.97 g, 18.58 mmol), and acetylacetone (1.91 g, 19.1 mmol) were added. The reaction mixture was added with 2-methoxyethanol (150 mL) and stirred to reflux at 125 °C for 8 h. After cooling to room temperature, the reaction mixture was filtered and the solid cake was washed with deionized water and anhydrous ethanol. The filter was dried and dissolved in dichloromethane. The crude product was purified by column chromatography. Deep-red solid (5.35 g) was obtained with a yield of 86.77%. Elemental analysis calculated for C_45_H_57_N_2_O_2_Ir: C, 64.34; H, 6.84; N, 3.33; found: C, 64.33; H, 6.83; N, 3.34. ^1^H-NMR (500 MHz, CDCl_3_) δ (ppm): 8.94–8.91 (d, J = 8.9 Hz, 2H), 8.25–8.23 (d, J = 6.4 Hz, 2H), 7.96 (s, 2H), 7.65–7.56 (m, 4H), 7.21–7.20 (d, J = 6.4 Hz, 2H), 6.53 (s, 2H), 4.86 (s, 1H), 3.16–3.12 (hept, J = 6.9 Hz, 2H), 2.31 (s, 6H), 1.53 (s, 6H), 1.53 (s, 6H), 1.41 (dd, J = 6.8, 3.2 Hz, 18H); ^13^C-NMR (125 MHz, CDCl_3_) δ (ppm): 184.41, 169.05, 151.08, 148.76, 146.89, 144.16, 140.89, 137.30, 131.21, 129.86, 128.12, 127.19, 126.79, 125.02, 123.27, 117.42, 99.72, 34.26, 28.43, 23.91, 23.79, 23.55, 21.22.

Ir(dmippiq)_2_(macac): The synthetic procedure was almost the same as Ir(dmippiq)_2_(acac), only using 3,5-heptone (2.45 g, 19.1 mmol) instead of acetylacetone. A deep-red solid (5.50 g) was obtained with a yield of 86.32%. Elemental analysis calculated for C_47_H_51_N_2_O_2_Ir: C, 65.02; H, 5.92; N, 3.23. found the following: C, 65.03; H, 5.91; N, 3.23. ^1^H NMR (500 MHz, CDCl_3_) δ(ppm): 8.92 (d, J = 8.9 Hz, 2H), 8.20 (d, J = 6.4 Hz, 2H), 7.96 (s, 2H), 7.62–7.50 (m, 4H), 7.18 (d, J = 6.4 Hz, 2H), 6.55 (s, 2H), 4.83 (s, 1H), 3.13 (hept, J = 7.0 Hz, 2H), 2.31 (d, J = 9.8 Hz, 6H), 1.84–1.68 (m, 4H), 1.46–1.30 (m, 18H), 0.37 (t, J = 7.6 Hz, 6H). ^13^C-NMR (125 MHz, CDCl_3_) δ(ppm): 189.40, 168.92, 151.01, 148.75, 146.82, 144.65, 140.95, 137.27, 131.00, 129.70, 128.03, 127.07, 126.76, 125.16, 123.12, 117.35, 97.09, 34.90, 34.22, 23.98, 23.79, 23.51, 21.23, 11.22.

Ir(dmippiq)_2_(dmacac): The synthetic procedure was almost the same as Ir(dmippiq)_2_(acac), only using 2,6-dimethyl-3,5-heptanedione (2.98 g, 19.1 mmol) instead of acetylacetone. A deep-red solid (5.40 g) was obtained with a yield of 82.09%. Elemental analysis calculated for C_49_H_55_N_2_O_2_Ir: C, 65.67; H, 6.19; N, 3.13. found the following: C, 65.65; H, 6.19; N, 3.12. ^1^H-NMR (500 MHz, CDCl_3_) δ (ppm): 8.90 (d, J = 8.8 Hz, 2H), 8.11 (t, J = 5.0 Hz, 2H), 7.94 (s, 2H), 7.61–7.47 (m, 4H), 7.14 (d, J = 6.4 Hz, 2H), 6.56 (s, 2H), 4.80 (s, 1H), 3.12 (hept, J = 6.9 Hz, 2H), 2.34 (s, 6H), 1.99 (hept, J = 6.8 Hz, 2H), 1.60–1.49 (m, 1H), 1.45–1.28 (m, 18H), 0.67 (d, J = 6.9 Hz, 6H), 0.33 (d, J = 6.8 Hz, 6H). ^13^C-NMR (125 MHz, CDCl_3_) δ (ppm): 192.33, 168.95, 150.90, 148.82, 146.76, 145.61, 140.86, 137.20, 130.58, 129.39, 127.93, 127.01, 126.71, 125.16, 123.04, 117.12, 94.10, 39.36, 34.22, 23.98, 23.82, 23.48, 21.25, 20.20, 19.19.

Ir(dmippiq)_2_(tmacac): The synthetic procedure was almost the same as Ir(dmippiq)_2_(acac), only using 2,2,6,6-tetramethyl-3,5-heptanedione (3.52 g, 19.1 mmol) instead of acetylacetone. A deep-red solid (5.60 g) was obtained with a yield of 82.60%. Elemental analysis calculated for C_51_H_59_N_2_O_2_Ir: C, 66.28; H, 6.43; N, 3.03. found the following: C, 66.26; H, 6.44; N, 3.03. ^1^H-NMR (500 MHz, CDCl_3_) δ (ppm):8.88–8.90 (d, J = 8.9 Hz, 2H), 8.10–8.08 (d, J = 6.4 Hz, 2H), 7.94 (s, 2H), 7.56–7.53 (m, 4H), 7.13–7.12 (d, J = 6.4 Hz, 2H), 6.55 (s, 2H), 5.12 (s, 1H), 3.15–3.09 (hept, J = 6.9 Hz, 2H), 2.34 (s, 6H), 1.44–1.37 (m, 18H), 1.36–1.16 (m, 2H), 0.63 (s, 18H), 0.03–−0.03 (m, 4H). ^13^C-NMR (125 MHz, CDCl_3_) δ (ppm): 193.71, 169.00, 150.81, 148.81, 146.75, 146.39, 140.94, 137.16, 130.34, 129.11, 127.85, 126.99, 126.65, 125.14, 122.99, 117.02, 88.60, 40.95, 34.21, 27.91, 23.99, 23.84, 23.46, 21.26.

Ir(dmippiq)_2_(ipacac): The synthetic procedure was almost the same as Ir(dmippiq)_2_(acac), only using diisovalerylmethane (3.52 g, 19.1 mmol) instead of acetylacetone. A deep-red solid (5.71 g) was obtained with a yield of 84.21%. Elemental analysis calculated for C_51_H_57_N_2_O_2_Ir: C, 66.42; H, 6.23; N, 3.04. found the following: C, 66.43; H, 6.22; N, 3.05. ^1^H-NMR (500 MHz, CDCl_3_) δ (ppm):8.91–8.89 (d, J = 8.8 Hz, 2H), 8.20–8.18 (d, J = 6.4 Hz, 2H), 7.96 (s, 2H), 7.59–7.56 (m, 4H), 7.18–7.16 (d, J = 6.4 Hz, 2H), 6.56 (s, 2H), 4.78 (s, 1H), 3.16–3.09 (hept, J = 6.8 Hz, 2H), 2.33 (s, 6H), 1.80–1.76 (dd, J = 12.2, 5.6 Hz, 2H), 1.58–1.50 (m, 2H), 1.44 (s, 6H), 1.40–1.37 (dd, J = 6.9, 2.6 Hz, 12H), 1.27–1.24 (m, 2H), 0.34–0.32 (d, J = 6.7 Hz, 6H), −0.11–−0.12 (d, J = 6.6 Hz, 6H). ^13^C-NMR (125 MHz, CDCl_3_) δ (ppm):186.81, 169.02, 151.07, 148.78, 146.84, 145.15, 140.95, 137.59, 130.98, 129.69, 128.11, 127.09, 126.66, 125.23, 123.08, 117.35, 100.96, 50.91, 34.27, 26.77, 23.98, 23.80, 23.61, 22.34, 21.24, 21.01.

Ir(dmippiq)_2_(deacac): The synthetic procedure was almost the same as Ir(dmippiq)_2_(acac), only using 3,7-diethylnonane-4,6-dione (4.06 g, 19.1 mmol) instead of acetylacetone. A deep-red solid (5.78 g) was obtained with a yield of 82.87%. Elemental analysis calculated for C_53_H_61_N_2_O_2_Ir: C, 66.99; H, 6.47; N, 2.95. found the following: C, 66.97; H, 6.45; N, 2.94. ^1^H-NMR (500 MHz, CDCl_3_) δ (ppm): 8.91–8.88 (d, J = 8.8 Hz, 2H), 8.17–8.15 (d, J = 6.4 Hz, 2H), 7.95 (s, 2H), 7.58–7.54 (m, 4H), 7.12–7.10 (d, J = 6.4 Hz, 2H), 6.56 (s, 2H), 4.80 (s, 1H), 3.12–3.08 (hept, J = 6.9 Hz, 2H), 2.34 (s, 6H), 1.56–1.52 (m, 2H), 1.44 (s, 6H), 1.39–1.37 (dd, J = 6.49, 3.5 Hz, 12H), 1.32–1.23 (m, 2H), 1.12–1.04 (m, 2H), 0.92–0.78 (m, 2H), 0.43–0.40 (t, J = 7.4 Hz, 6H), −0.17–−0.19 (t, J = 7.4 Hz, 6H). ^13^C-NMR (125 MHz, CDCl_3_) δ (ppm): 189.54, 169.03, 150.94, 148.81, 146.87, 145.94, 141.16, 137.63, 130.54, 129.41, 127.98, 127.05, 126.60, 125.26, 123.02, 116.90, 100.44, 54.89, 34.27, 26.53, 26.16, 23.99, 23.78, 23.59, 21.26, 11.68, 11.17.

Ir(dmippiq)_2_(dmeacac): The synthetic procedure was almost the same as Ir(dmippiq)_2_(acac), only using 3,7-dimethyl-4,6-nonanedione (3.52 g, 19.1 mmol) instead of acetylacetone. A deep-red solid (5.48 g) was obtained with a yield of 80.78%. Elemental analysis calculated for C_51_H_59_N_2_O_2_Ir: C, 66.28; H, 6.43; N, 3.03. found the following: C, 66.30; H, 6.44; N, 3.04. ^1^H-NMR (500 MHz, CDCl_3_) δ (ppm): 8.90–8.89 (dd, J = 8.3, 5.4 Hz, 2H), 8.15–8.11 (m, 2H), 7.95 (s, 2H), 7.59–7.54 (m, 3H), 7.14–7.12 (dd, J = 6.5, 1.5 Hz, 2H), 6.57 (s, 2H), 4.79–4.76 (t, J = 6.1 Hz, 1H), 3.13–3.10 (hept, J = 6.9 Hz, 2H), 2.35 (s, 6H), 1.82–1.71 (m, 2H), 1.63–1.48 (m, 1H), 1.46–1.45 (d, J = 2.6 Hz, 6H), 1.39–1.37 (dd, J = 6.9, 3.6 Hz, 12H), 1.26–1.14 (m, 2H), 1.02–0.95 (m, 2H), 0.78–0.76 (m, 6H), 0.36–0.28 (ddd, J = 14.9, 11.0, 6.4 Hz, 6H), 0.20–0.24 (td, J = 7.4, 3.1 Hz, 4H). ^13^C-NMR (125 MHz, CDCl_3_) δ (ppm): 191.34, 191.30, 190.79, 169.06, 169.00, 150.95, 150.92, 148.82, 148.78, 146.84, 146.80, 145.91, 145.82, 145.77, 140.90, 140.87, 137.49, 137.34, 137.32, 130.60, 130.49, 129.46, 129.44, 129.34, 127.95, 127.05, 126.65, 126.63, 125.23, 123.02, 117.05, 117.02, 116.99, 98.26, 97.23, 95.88, 46.95, 46.88, 46.81, 46.77, 34.27, 34.25, 27.70, 27.66, 27.21, 27.18, 23.99, 23.83, 23.80, 23.58, 23.52, 21.27, 18.69, 18.67, 17.49.

## 4. Conclusions

In conclusion, a series of HICs with different β-diketone ancillary ligands were designed and synthesized. By engineering the alkyl group substitute of β-diketone, systematic investigations of thermal, optical, and electrochemical properties of the seven HICs were conducted, revealing that alkyl group of β-diketone have little influence on their basic characteristics. However, Ir(dmippiq)_2_(deacac) exhibited more ordered molecular packing and strong molecular interactions as its thin film state. By fabrications of the corresponding PhOLEDs, all the devices gave a deep-red emission with a EL peak at around 624 nm and CIE coordinates of (0.68, 0.32). With a doping concentration of 16%, the Ir(dmippiq)_2_(deacac)-based device achieved a champion device performance, with a L_max_ of 13,960 cd/m^2^, a CE_max_ of 15.63 cd/A, a PE_max_ of 12.60 lm/W, and an EQE_max_ of 18.26%. From the perspective of ancillary ligand engineering, our results demonstrate that β-diketone can play an important role in PhOLED device performance and provide a design.

## Figures and Tables

**Table 1:** Crystal data of Ir(dmippiq)_2_(deacac).

Complexes	Ir(dmppiq)_2_(dpacac)
Formula	C_53_H_63_IrN_2_O_2_
Formula weight	952.25
Crystal system	monoclinic
Space group	P121/c1
*a* [Å]	15.9566(13)
*b* [Å]	14.4918(12)
*c* [Å]	40.128(3)
*α* [°]	90°
*β* [°]	90.850(2)°
*γ* [°]	90°
*V* [Å^3^]	9278.2(13)
Z	8
*ρ*_calc_ [g cm^−3^]	1.363
Reflections collected	90,736
Independent reflections	15,975
R, WR_2_(all)	0.0943, 0.1711
R, WR_2_ [I > 2σ(I)]	0.0599, 0.1546
GOF	1.078

**Table 2:** Selected bond length (Å) and bond angle (°) for Ir(dmppiq)_2_(dpacac).

Compound	Ir(dmppiq)_2_(dpacac)		
Ir(1)-O(1)	2.128(5)	Ir(1)-O(2)	2.130(5)
Ir(1)-N(1)	2.032(6)	Ir(1)-N(2)	2.044(6)
Ir(1)-C(16)	2.007(7)	Ir(1)-C(36)	2.006(8)
O(1)-Ir(1)-O(2)	88.6(2)	C(16)-Ir(1)-N(1)	80.3(3)
C(36)-Ir(1)-N(2)	80.1(3)	N(1)-Ir(1)-N(2)	174.1(2)
C(16)-Ir(1)-O(1)	174.5(3)	C(36)-Ir(1)-O(2)	175.2(3)

**Table 3:** Summary of electronic, optical, and thermal properties.

Compound	λ_abs_ ^a^ (nm)	λ_ex_ ^a^ (nm)	λ_PL_ ^a^ (nm)	Ф_PL_ ^a^	τ ^b^ (ns)	k_r_ ^b^ (×10^6^ s^−1^)	k_nr_ ^b^ (×10^6^ s^−1^)	T_d_ (°C)
Ir(dmippiq)_2_(acac)	295(6.86), 348(3.56), 424(2.16), 479(1.32)	281	620	0.74	111.32	6.65	2.34	399
Ir(dmippiq)_2_(macac)	296(7.41), 349(3.67), 425(1.96), 477(1.21)	285	620	0.75	117.48	6.38	2.13	357
Ir(dmipiq)_2_(dmacac)	295(8.23), 347(3.96), 426(2.40), 478(1.35)	273	621	0.68	144.97	4.69	2.21	364
Ir(dmippiq)_2_(tmacac)	294(6.58), 348(3.14), 429(1.87), 480(1.04)	281	623	0.72	209.05	3.44	1.34	355
Ir(dmippiq)_2_(ipacac)	294(7.36), 348(3.05), 426(2.20), 478(1.25)	277	619	0.70	157.04	4.46	1.91	377
Ir(dmippiq)_2_(deacac)	294(7.23), 348(3.45), 428(2.16), 478(1.24)	277	618	0.66	198.55	3.32	1.71	343
Ir(dmipiq)_2_(dmeacac)	295(7.63), 348(3.79), 427(2.41), 478(1.44)	287	619	0.72	164.53	4.38	1.70	333

^a^ λ_abs_, λ_ex_, λ_PL_, Ф_PL_ measured in DCM solution. and molar extinction coefficients (10^4^ M^−1^ cm^−1^) are shown in parentheses ^b^ Radiative decay rate k_r_ = Φ_PL_/τ, and non-radiative decay rate k_nr_ = (1 − Φ_PL_)/τ.

**Table 4:** Summary of electronic properties.

Compound	E_ox_(Ir^3+^/Ir^4+^) (V)	E_g_ ^a^ (eV)	HOMO ^b^ (eV)	LUMO ^b^ (eV)
Ir(dmippiq)_2_(acac)	0.55	1.99	−4.95	−2.96
Ir(dmippiq)_2_(macac)	0.52	2.01	−4.92	−2.91
Ir(dmippiq)_2_(dmacac)	0.51	1.98	−4.91	−2.93
Ir(dmippiq)_2_(tmacac)	0.48	1.99	−4.88	−2.89
Ir(dmippiq)_2_(ipacac)	0.55	1.99	−4.95	−2.96
Ir(dmippiq)_2_(deacac)	0.52	1.99	−4.92	−2.93
Ir(dmippiq)_2_(dmeacac)	0.51	1.98	−4.91	−2.93

^a^ E_g_ was calculated from the cross-point of absorption and PL spectrum with an equation of E_g_ = 1240/λ eV. ^b^ the HOMO and LUMO values were determined using the following equations: E_HOMO_ (eV) = −e(E_ox_+ 4.4) eV, E_LUMO_ (eV) = E_HOMO_ + E_g_.

**Table 5:** EL performance of the optimized doping concentration of each HIC.

HIC	Doping Concentration	*L*_max_ ^a^ (cd/m^2^)	CE_max_ ^b^ (cd/A)	PE_max_ ^c^ (lm/W)	EQE ^d^ (%)	*V*_D_ (V) @ 1, 100, 1000 cd/m^2^	EL Peak ^e^ (nm)	CIE (x, y)
Ir(dmippiq)_2_(acac)	12%	4651	7.49	5.30	14.80	4.3, 7.7, 10.2	624	(0.68, 0.32)
Ir(dmippiq)_2_(macac)	10%	7026	7.55	3.95	13.42	5.9, 7.9, 9.6	624	(0.68, 0.32)
Ir(dmippiq)_2_(dmacac)	10%	8355	7.71	4.03	13.31	5.8, 7.8, 9.5	624	(0.68, 0.32)
Ir(dmippiq)_2_(tmacac)	12%	9973	10.89	8.77	13.45	2.7, 3.9, 5.7	625	(0.68, 0.32)
Ir(dmippiq)_2_(ipacac)	10%	9288	11.03	8.89	13.29	2.7, 3.8, 5.7	626	(0.68, 0.32)
Ir(dmippiq)_2_(deacac)	10%	5024	11.77	9.48	13.49	2.9, 5.3, 8.4	624	(0.68, 0.32)
Ir(dmippiq)_2_(dmeacac)	16%	13,960	15.63	12.60	18.26	2.6, 3.6, 5.4	625	(0.68, 0.32)

^a^ *L*_max_: maximum luminance. ^b^ CE_max_: maximum current efficiency. ^c^ PE_max_: maximum power efficiency. ^d^ EQE_max_: maximum external quantum efficiency. ^e^ EL peak: the wavelength where the EL spectrum has the maximum intensity.

## Data Availability

The data presented in this study are contained within the article. The crystallographic data have been deposited in the Cambridge Crystallographic Data Centre (CCDC No: 2413035). The NMR spectrum and performance data of devices with different doping concentrations are included in Supplementary Materials.

## Supplementary Materials

The following supporting information can be downloaded at: https://www.mdpi.com/article/10.3390/molecules30040861/s1, ^1^H NMR, ^13^C NMR, fluorescence lifetimes and data related to device fabrication and optimization of HICs.
