# Supplementary material for: Synthesis, Photo-Physical Properties, and Electroluminescence Characteristics of Iridium Phosphorescent Materials Based on Different β-Diketonate Ancillary Ligands
Source: Molecules. 2025 Feb 13;30(4):861. doi: 10.3390/molecules30040861 (PMC11858730; doi:10.3390/molecules30040861)
Supplement: Supplementary file 1 [file molecules-30-00861-s001.zip › molecules-3427056-supplementary.pdf]

Supplementary Information

Ancillary ligand engineering of alkyl groups in deep red-emitting phosphorescent materials for efficient organic light-emitting devices

Qiao-Wen Chang [1,*], Caixian Yan [1], Honghui Wei [2], Jian Yang [3], Liming Xie [2,4], Yuan-Qiu-Qiang Yi [2,3,*], Wenming Su [2,3], Weiping Liu [1]

[1] State Key Laboratory of Advanced Technologies for Comprehensive Utilization of Platinum Metals, Kunming Institute of Precious Metals, Kunming 650000, China
[2] Printable Electronics Research Center, Nano-Devices and Materials Division, Suzhou Institute of Nano-Tech and Nano-Bionics, Chinese Academy of Sciences, Suzhou 215123, Jiangsu, China
[3] Gusu Laboratory of Materials, Suzhou 215123, Jiangsu, China
[4] Joint International Research Laboratory of Information Display and Visualization, School of Electronic Science and Engineering, Southeast University, Nanjing 210096, China

Corresponding author e-mail: changqiaowen@126.com, yqqyi2020@sinano.ac.cn

Ir(dmippiq)$_2$(acac)

^{1}H NMR

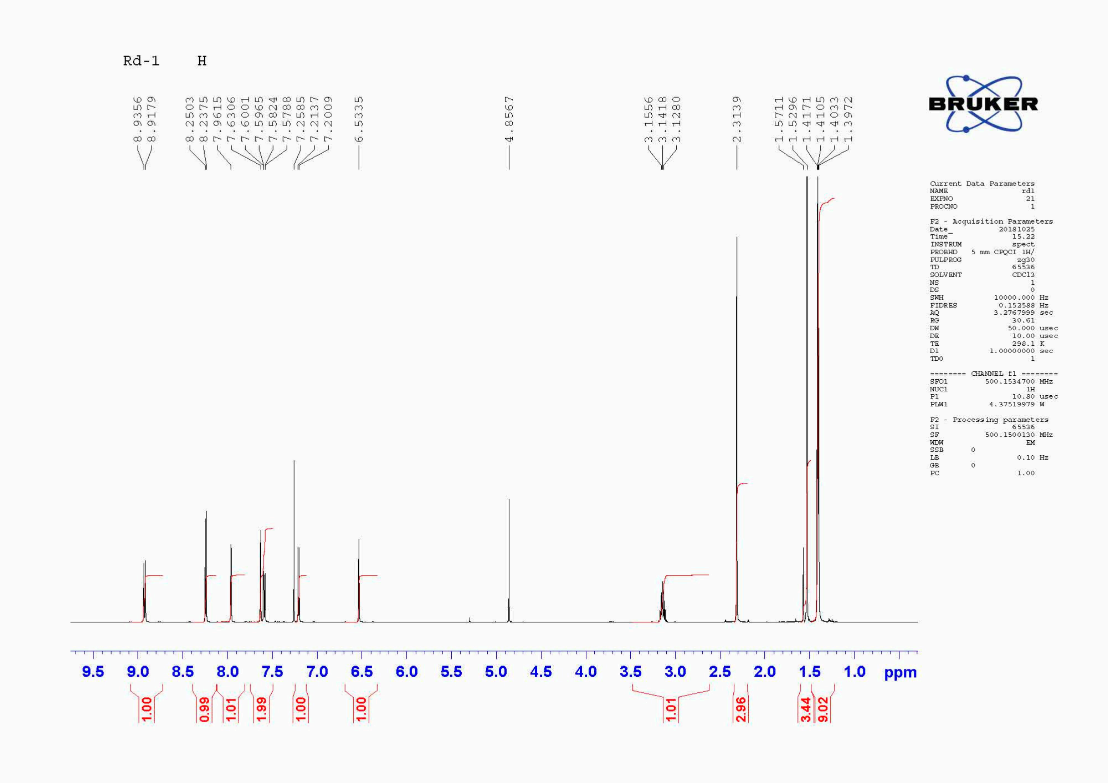

^{13}C NMR

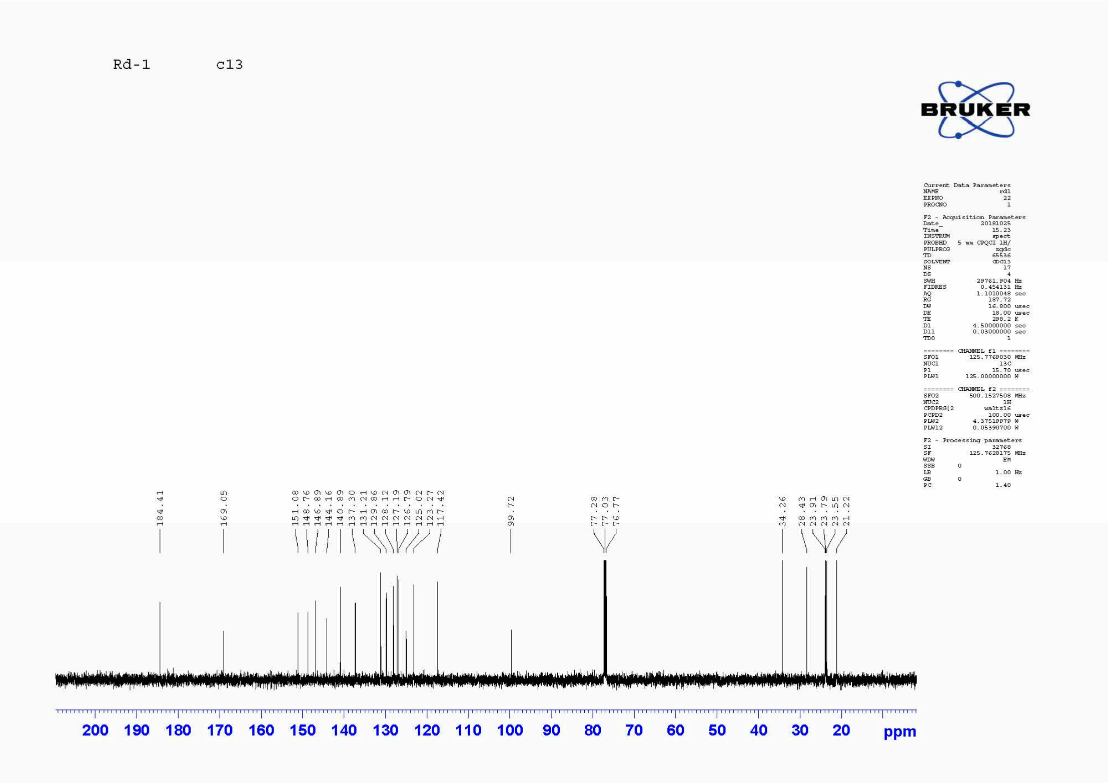

Ir(dmippiq)$_2$(macac)

^{1}H NMR

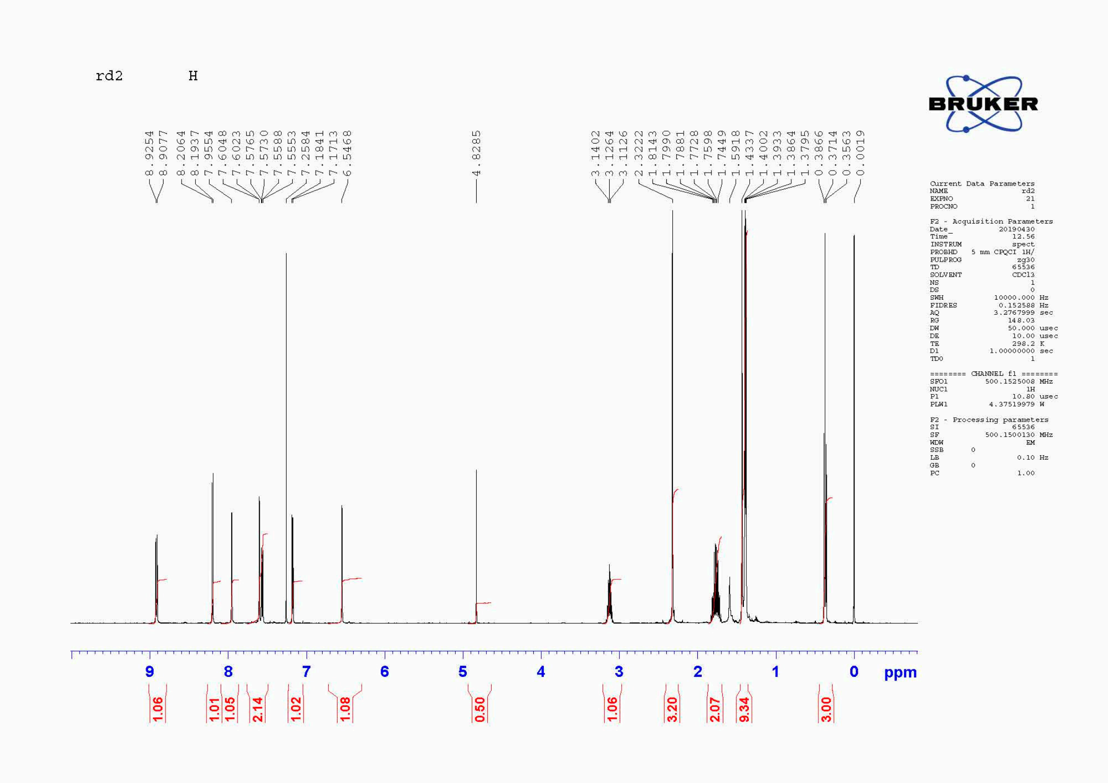

^{13}C NMR

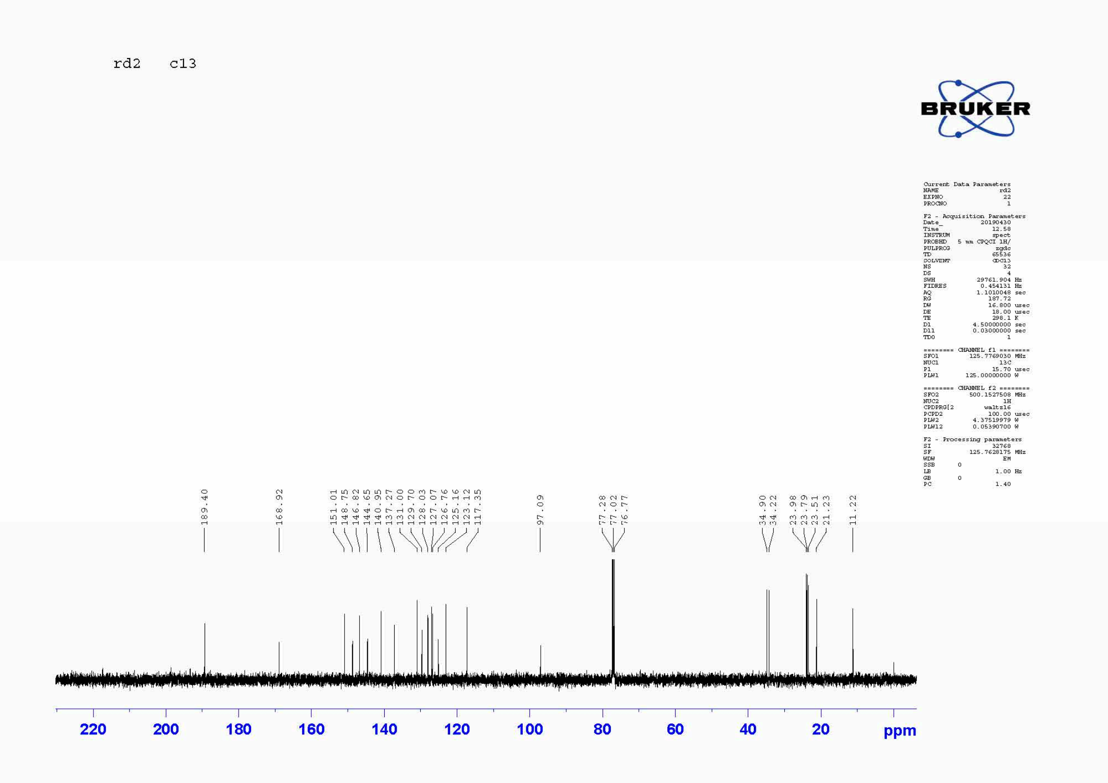

Ir(dmippiq)$_2$(dmacac)

^{1}H NMR

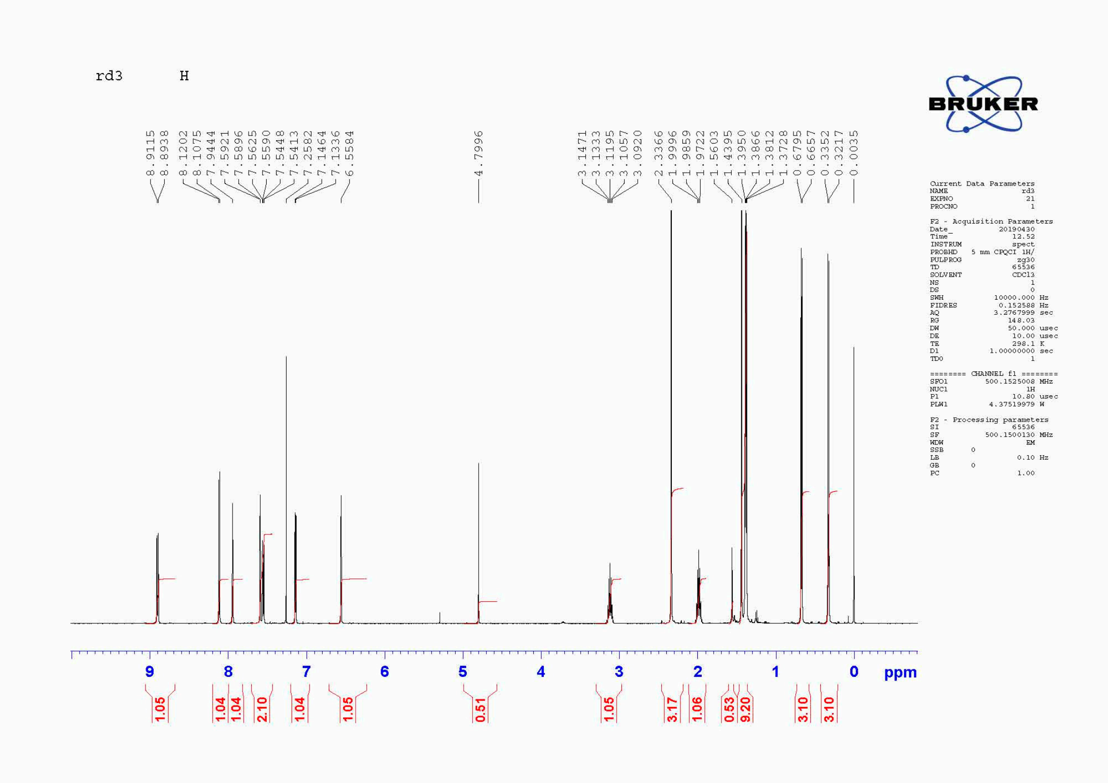

^{13}C NMR

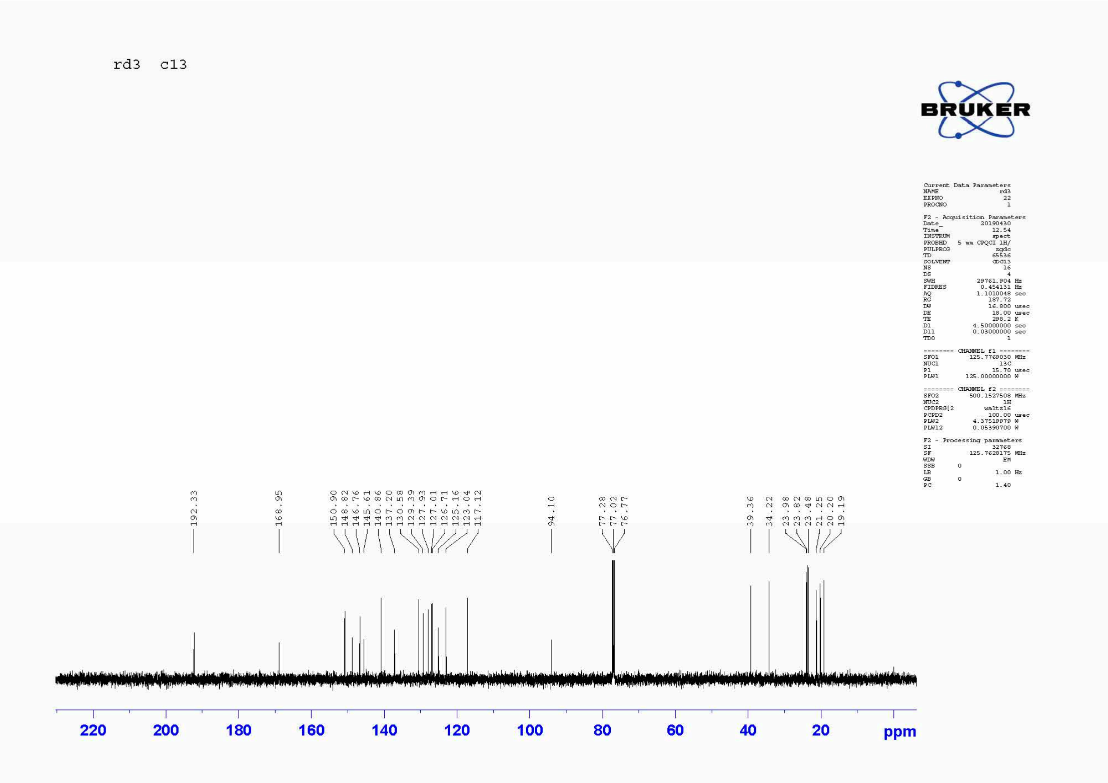

Ir(dmippiq)$_2$(tmacac)

^{1}H NMR

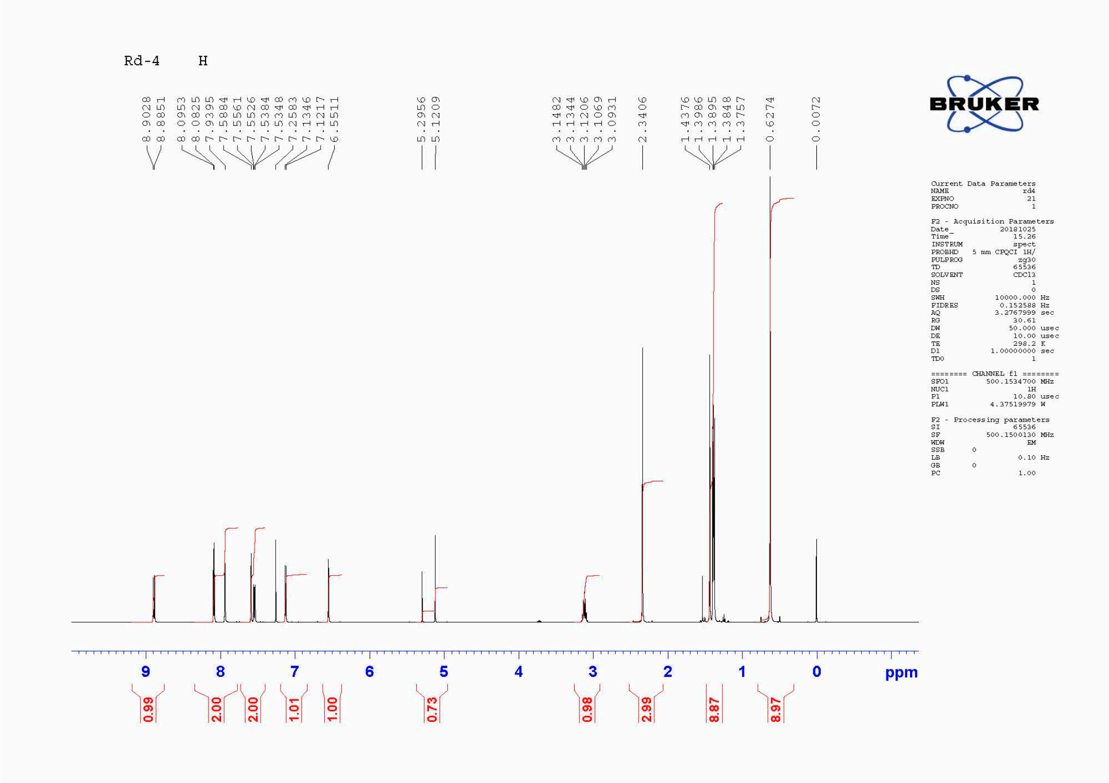

^{13}C NMR

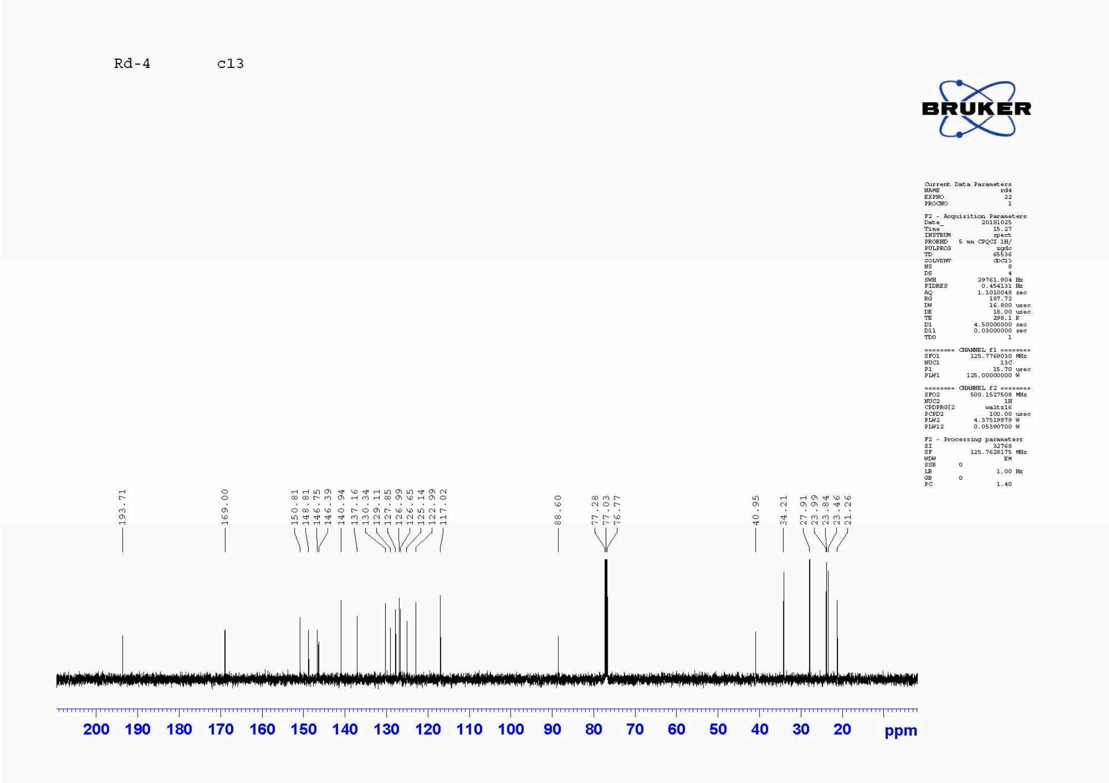

Ir(dmippiq)$_2$(ipacac)

^{1}H NMR

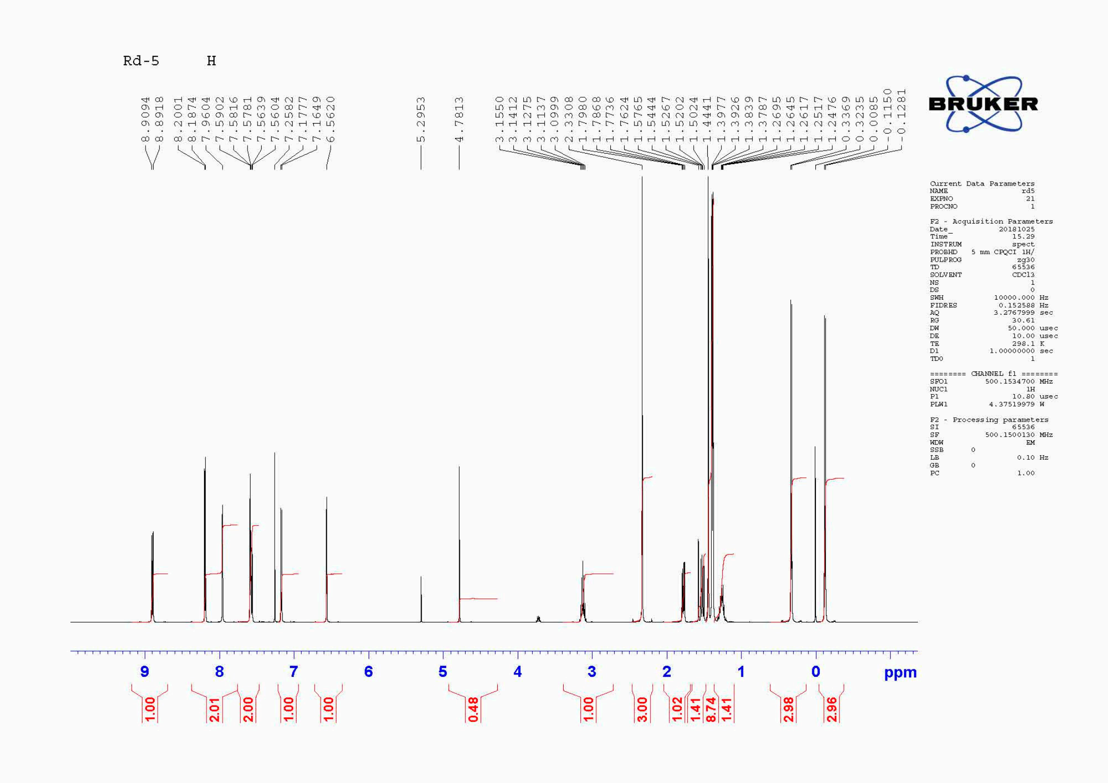

^{13}C NMR

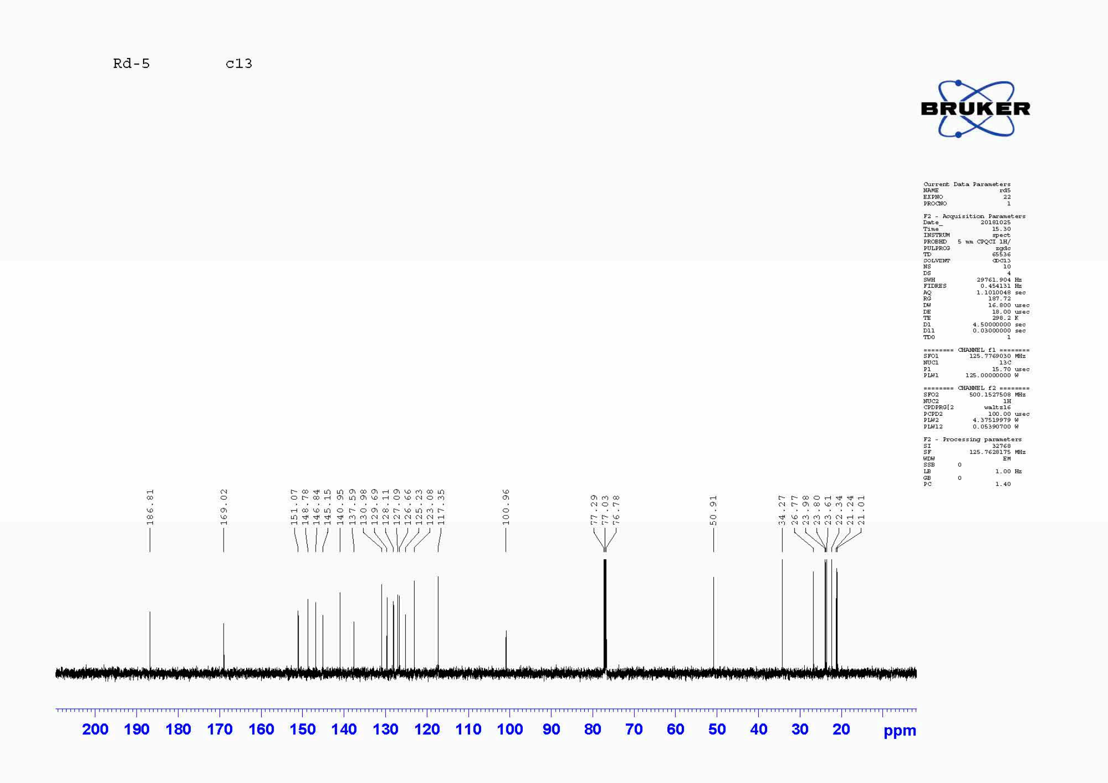

Ir(dmippiq)$_2$(deacac)

^{1}H NMR

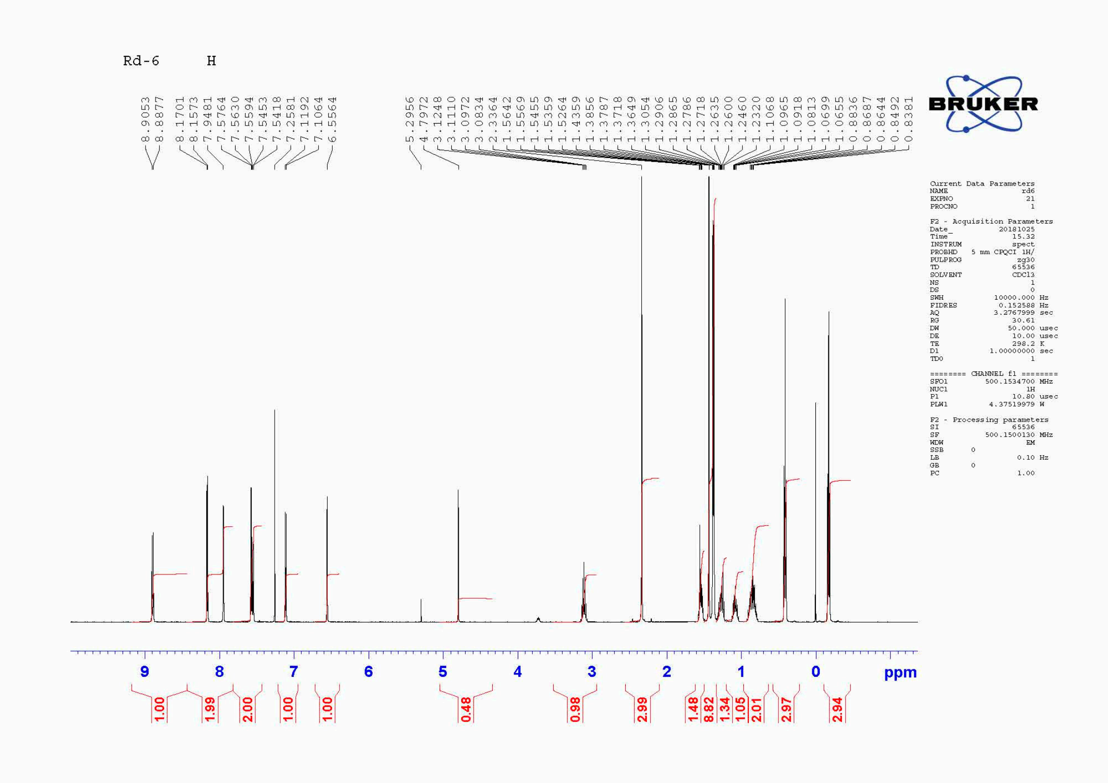

^{13}C NMR

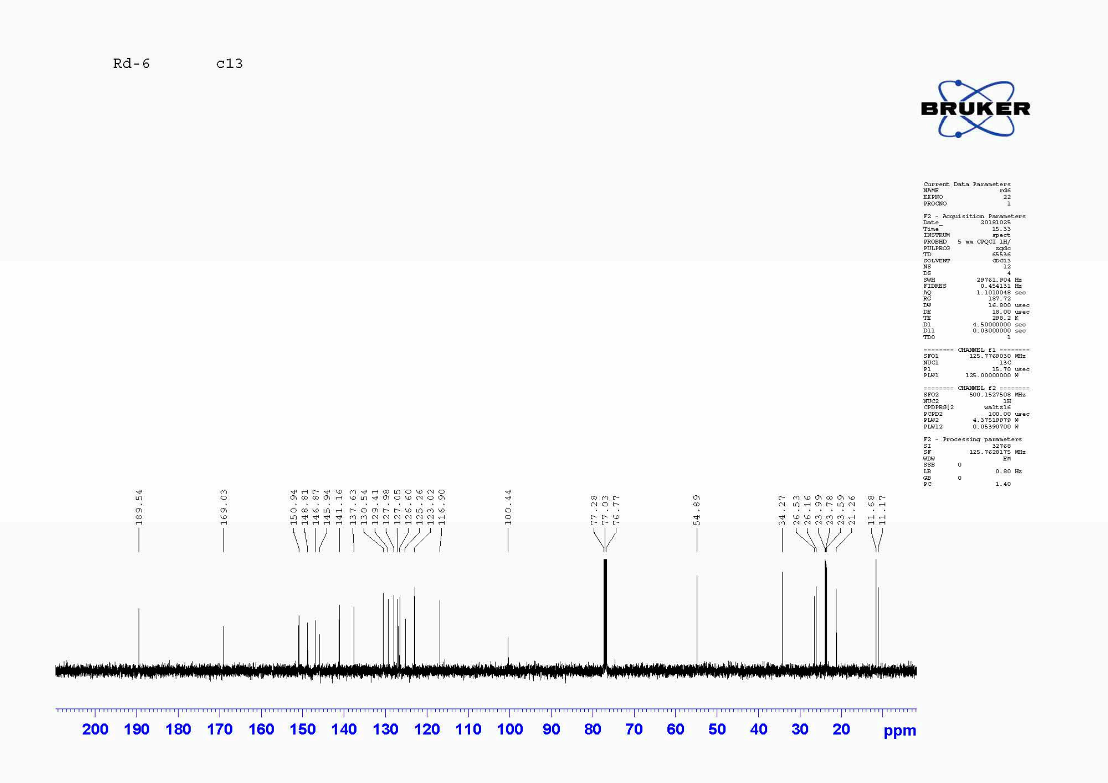

Ir(dmippiq)$_2$(dmeacac)

^{1}H NMR

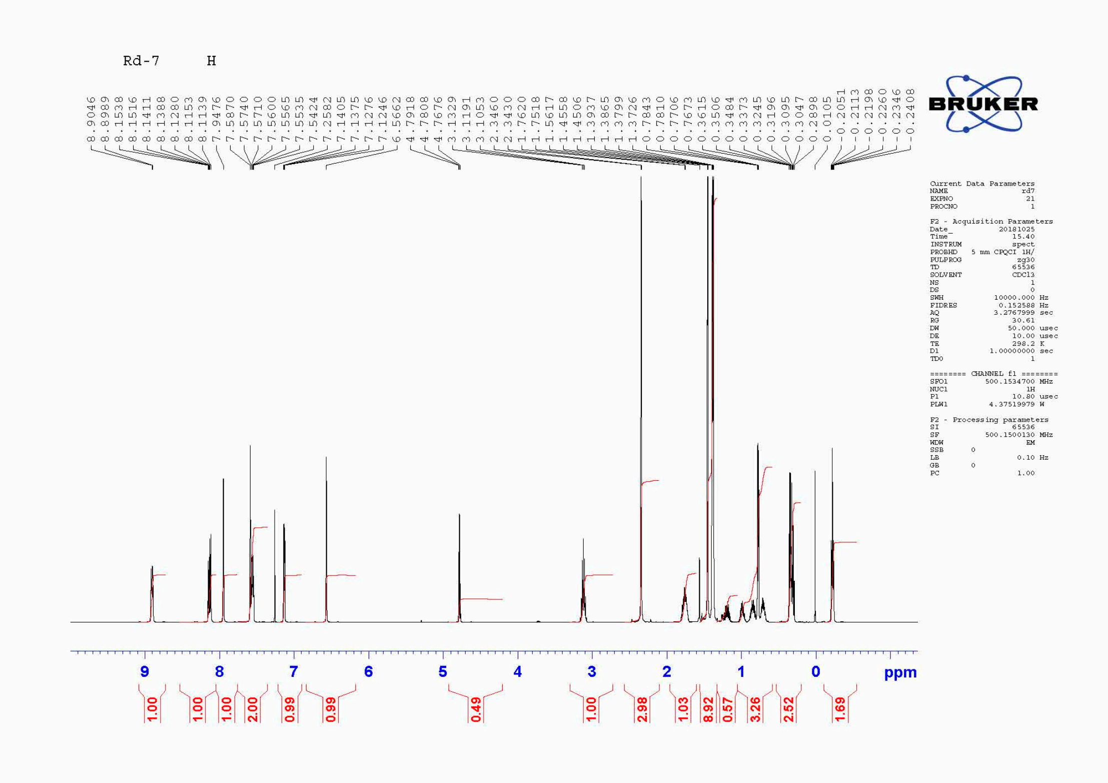

^{13}C NMR

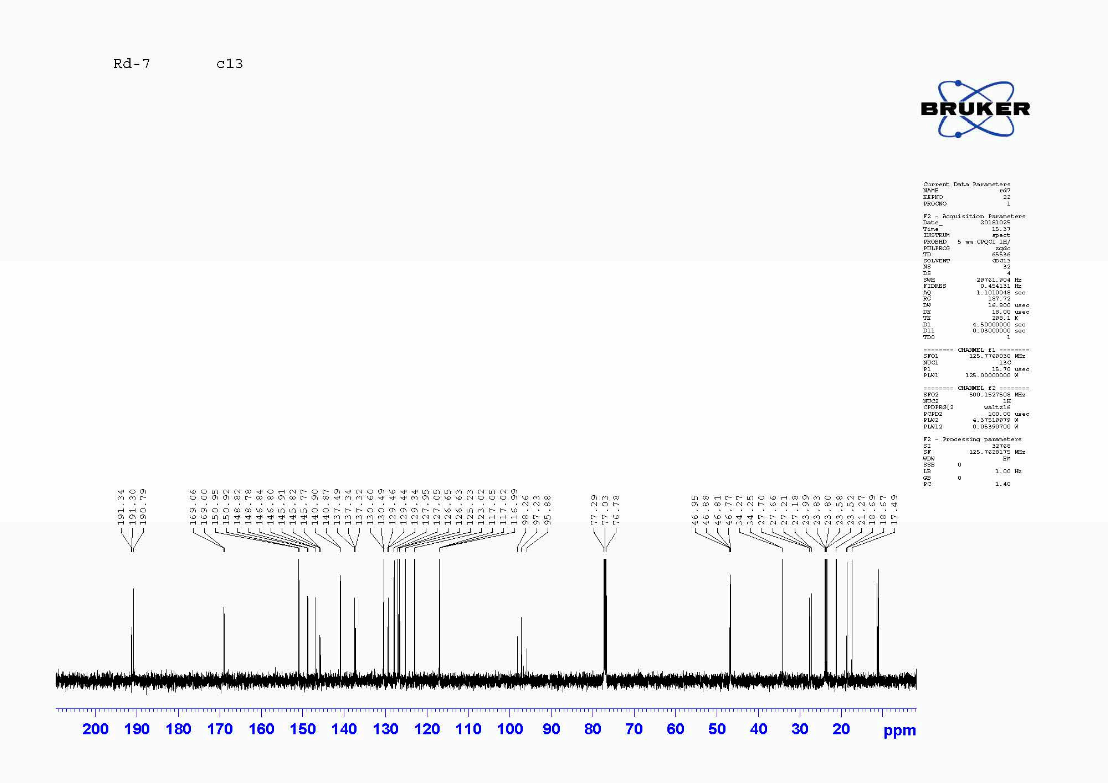

Fluorescent lifetime

Ir(dmippiq)$_2$(acac):

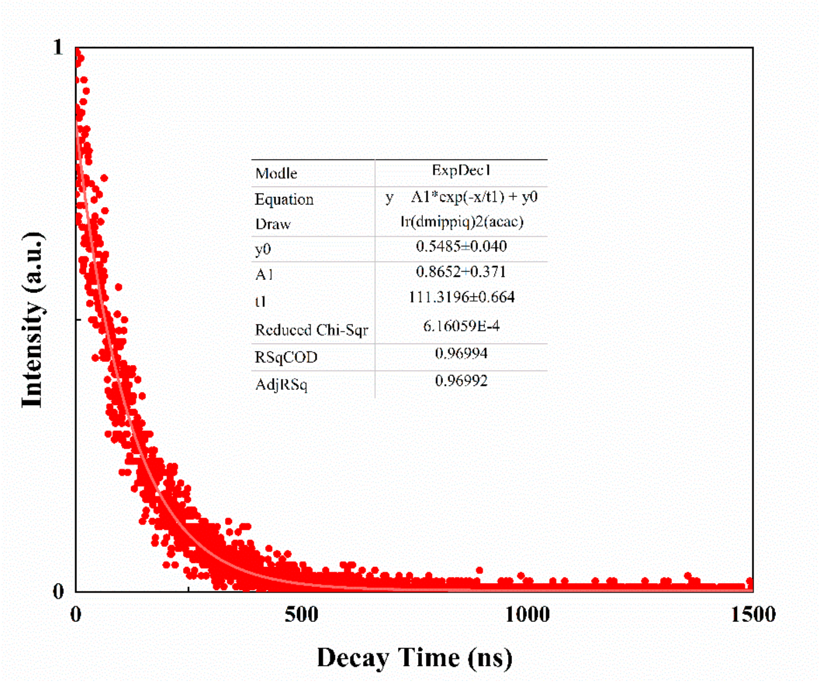

Ir(dmippiq)$_2$(macac):

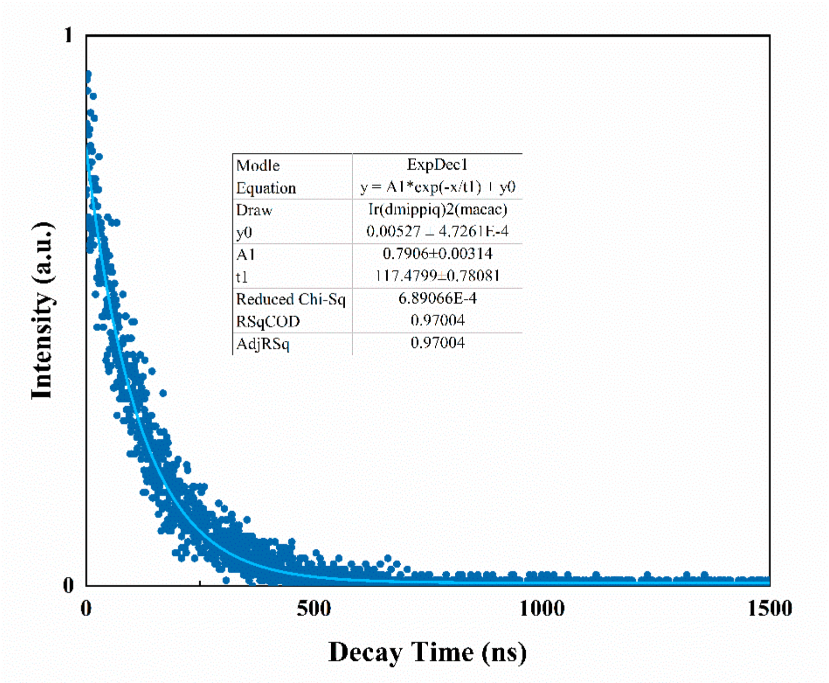

Ir(dmippiq)$_2$(dmacac):

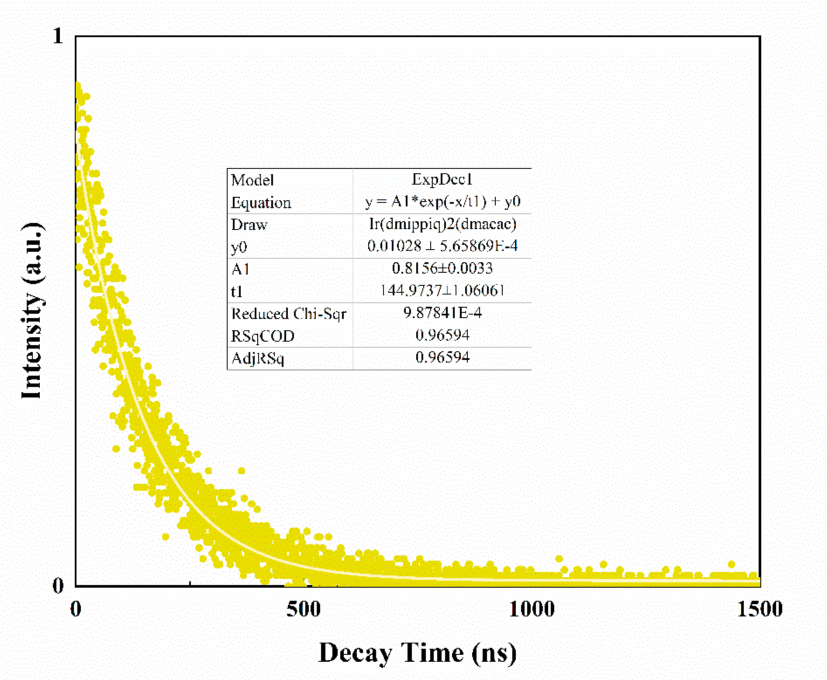

Ir(dmippiq)$_2$(tmacac):

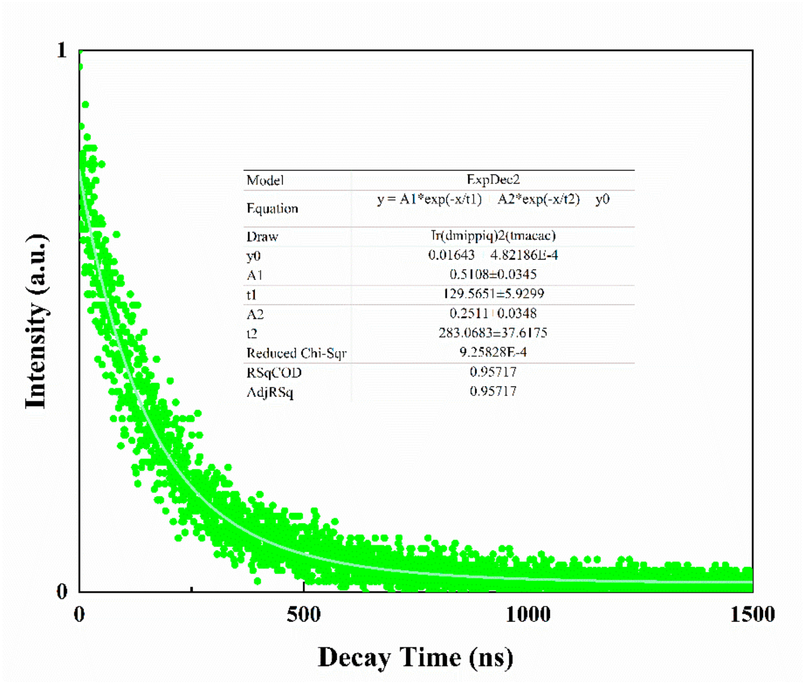

Ir(dmippiq)$_2$(ipacac):

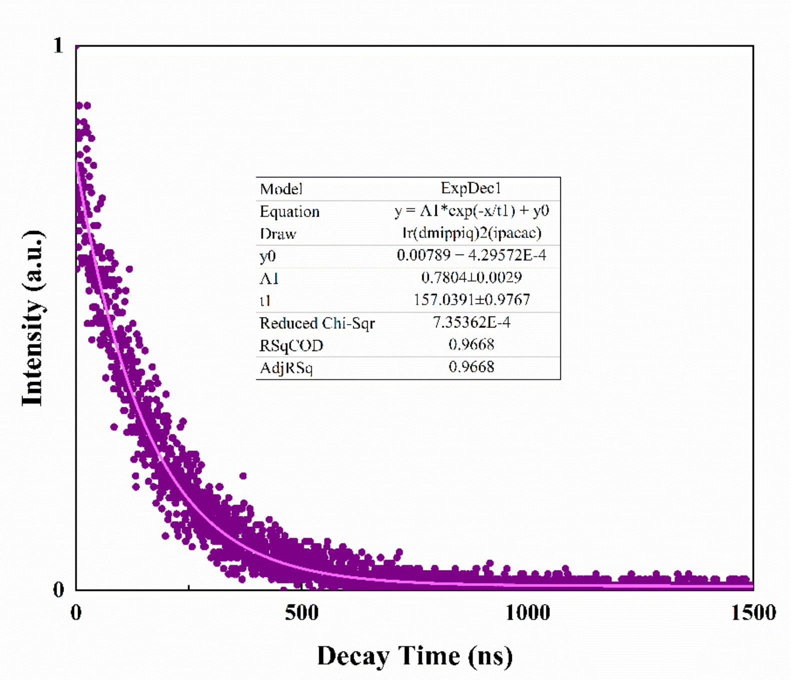

Ir(dmippiq)$_2$(deacac):

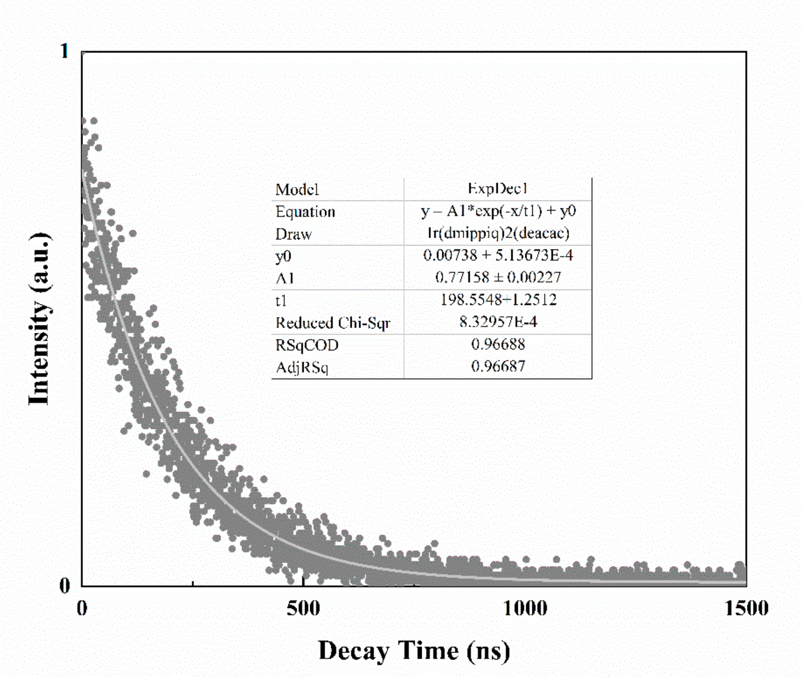

Ir(dmippiq)$_2$(dmeacac):

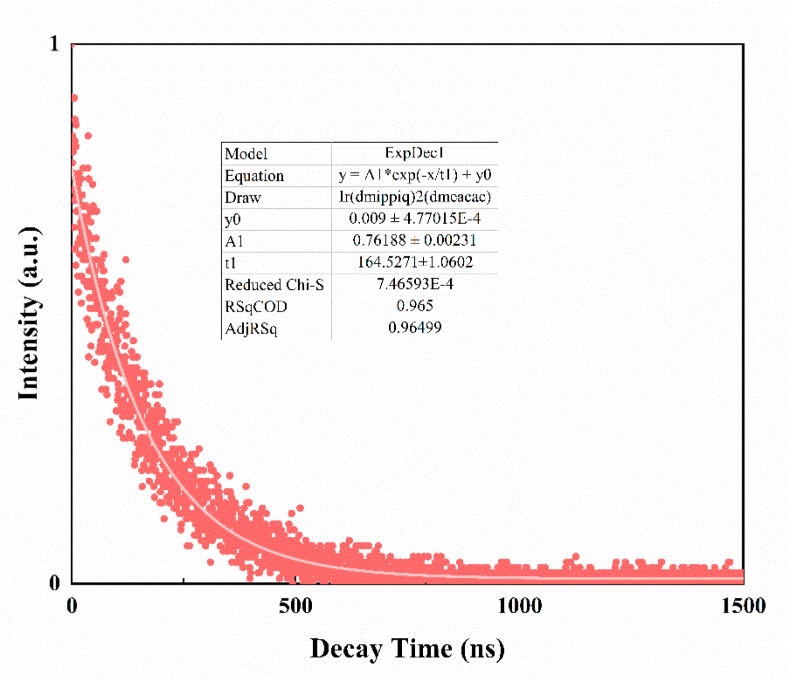

Device fabrications and optimizations of Ir(III) complexes

Ir(dmippiq)$_2$(acac):

Doping concentration (%)	L_{max} (cd/m^2)	CE_{max} (cd/A)	PE_{max} （lm/W）	EQEmax (%)	EL peak (nm)	CIE (x,y)
8 %	6619	6.29	2.81	9.15	624	0.68, 0.32
10 %	8355	7.71	4.03	13.31	624	0.68, 0.32
12 %	8550	6.60	2.94	9.95	624	0.68, 0.32
14 %	8660	7.40	3.80	11.93	624	0.68, 0.32

Ir(dmippiq)$_2$(macac):

Doping concentration (%)	L_{max} (cd/m^2)	CE_{max} (cd/A)	PE_{max} (lm/W)	EQE_{max} (%)	EL peak (nm)	CIE (x,y)
8 %	6151	6.27	3.28	11.42	624	0.68, 0.32
10 %	7026	7.55	3.95	13.42	624	0.68, 0.32
12 %	6781	7.51	3.93	12.40	624	0.68, 0.32
14 %	7454	6.13	2.37	9.66	624	0.68, 0.32

Ir(dmippiq)$_2$(dmacac):

Doping concentration (%)	L_{max} (cd/m^2)	CE_{max} (cd/A)	PE_{max} (lm/W)	EQE_{max} (%)	EL peak (nm)	CIE (x,y)
8 %	6619	6.29	2.81	9.15	624	0.68, 0.32
10 %	8355	7.71	4.03	13.31	624	0.68, 0.32
12 %	8550	6.60	2.94	9.95	624	0.68, 0.32
14 %	8660	7.40	3.80	11.93	624	0.68, 0.32

Ir(dmippiq)$_2$(tmacac):

Doping concentration (%)	L_{max} (cd/m^2)	CE_{max} (cd/A)	PE_{max} (lm/W)	EQE_{max} (%)	EL peak (nm)	CIE (x,y)
8 %	10890	10.20	8.22	12.47	624	0.68, 0.32
10 %	9950	11.10	8.95	13.12	625	0.68, 0.32
12 %	9973	10.89	8.77	13.45	625	0.68, 0.32
14 %	12510	8.98	10.45	11.72	626	0.68, 0.32

Ir(dmippiq)$_2$(ipacac):

Doping concentration (%)	L_{max} (cd/m^2)	CE_{max} (cd/A)	PE_{max} (lm/W)	EQE_{max} (%)	EL peak (nm)	CIE (x,y)
8 %	3334	8.80	6.59	9.48	624	0.68, 0.32
10 %	9288	11.03	8.89	13.29	626	0.68, 0.32
12 %	7079	9.29	6.95	11.36	626	0.68, 0.32
14 %	8377	9.51	9.05	11.75	626	0.68, 0.32

Ir(dmippiq)$_2$(deacac):

Doping concentration (%)	L_{max} (cd/m^2)	CE_{max} (cd/A)	PE_{max} (lm/W)	EQE_{max} (%)	EL peak (nm)	CIE (x,y)
8 %	5421	11.39	8.52	13.11	624	0.68, 0.32
10 %	5024	11.77	9.48	13.49	624	0.68, 0.32
12 %	5403	11.59	8.09	13.19	624	0.68, 0.32
14 %	5251	10.45	10.95	12.44	624	0.68, 0.32

Ir(dmippiq)$_2$(dmeacac):

Doping concentration (%)	L_{max} (cd/m^2)	CE_{max} (cd/A)	PE_{max} (lm/W)	EQE_{max} (%)	EL peak (nm)	CIE (x,y)
8 %	8956	7.41	7.06	8.39	624	0.68, 0.32
10 %	10110	10.36	9.55	11.77	624	0.68, 0.32
12 %	8837	10.57	10.99	12.23	624	0.68, 0.32
14 %	4279	11.44	11.44	12.71	624	0.68, 0.32
16 %	13960	15.63	12.60	18.26	625	0.68, 0.32
18 %	12090	15.39	12.40	18.15	625	0.68, 0.32
20 %	6822	15.23	15.95	18.30	625	0.68, 0.32
22 %	14170	14.87	12.98	17.99	625	0.68, 0.32
